# Research on the evolution of college online public opinion risk based on improved Grey Wolf Optimizer combined with LSTM model

**DOI:** 10.1371/journal.pone.0311749

**Published:** 2025-01-24

**Authors:** Chao Cao, Ziyu Li, Lingzhi Li, Fanglu Luo

**Affiliations:** 1 School of Marxism, Central South University, Changsha, China; 2 Student Work Department, Central South University, Changsha, China; 3 School of Public Administration, Central South University, Changsha, China; 4 School of Mechanical and Electrical Engineering, Central South University, Changsha, China; Akita University: Akita Daigaku, JAPAN

## Abstract

Since the dissemination of information is more rapid and the scale of users on online platforms is enormous, the public opinion risk is more visible and harder to tackle for universities and authorities. Improving the accuracy of predictions regarding online public opinion crises, especially those related to campuses, is crucial for maintaining social stability. This research proposes a public opinion crisis prediction model that applies the Grey Wolf Optimizer (GWO) algorithm combined with long short-term memory (LSTM) and implements it to analyze a trending topic on Sina Weibo to validate its prediction accuracy. A full-chain analytical framework for online public opinion prediction is established in this study, which enables the model to illustrate the level of risk related to public opinion and its variation trend by introducing the public opinion risk index. The prediction accuracy of the model is validated through several evaluation criteria, and a comparison between real and predicted results, and the simulation of the intervention on this incident indicates that the proposed model is competent for both trend prediction and assisting in intervention. The study also demonstrates the importance of immediate response and intervention to public opinion crises.

## 1. Introduction

With the rapid development and popularization of network information and intelligent technology, the Internet not only embodies technical attributes but is also endowed with communication attributes, social attributes, and ideological attributes. Public opinion is formed by people’s dialogues, comments, etc., which can be spread and known to numerous people on online platforms. Various ideas and opinions are expressed through network platforms and are disseminated quickly, making it extremely easy to form a "field of public opinion" that is detrimental to the security and stability of society, which makes it necessary to research public opinion hotspots. Colleges and universities are active in the ideological field and have always been the focus of social attention. Teachers and university students have active minds and a strong connection to the Internet. Therefore, it is easy for crises of public opinion to emerge on campuses, as we can see in the fast escalating rat head and duck neck incident in China [1].

Public opinion in colleges, as a kind of public opinion disseminated through a network, has particularities. First of all, the audience is broad, as it not only encompasses teachers and students but also alumni, parents, the public, and other groups. The spread of public opinion from colleges is therefore wide and has great influence, increasing the likelihood of an evolution into a social crisis. Secondly, the sensitivity is high. The public opinion of colleges and universities—as educational institutions—is often closely related to tertiary education, academic research, and student management. Once a negative public opinion appears, people will allocate part of their attention to the related institutions inevitably and it may have a serious impact on the reputation, enrollment, and stability of the faculty and staff of a school. The public opinion of colleges and universities often spreads rapidly in a short time, which poses great challenges to their reputation and image.

However, colleges usually lack preparation for monitoring and managing public opinion and crises; they find themselves in a passive position and miss the chance within the "golden hours" [2] to stabilize the situation. Preparing for such situations requires colleges to discover the risks related to online public opinion in time, monitor the trends in public opinion on and off campus, discover potential crises, grasp the trends in the development of public opinion over time, and then predict possible crises in advance in order to launch coping strategies and reduce the impacts of such crises on schools.

Strengthening the governance of the online public opinion of colleges is necessary not only for maintaining campus security and a good reputation but also for providing an academic atmosphere for education and nurturing talent on campuses. In this research, we will further clarify the mechanisms underlying the occurrence of online public opinion in universities according to their characteristics and propose effective management and control strategies.

According to Cai et al. [3], online platforms like X (Twitter) and Weibo have the characteristics of rapid dissemination, vast influence, and robust interactivity, which is obviously different from traditional media and has a great impact on online public opinion. Due to the complexity, rapidity, suddenness, and interactivity of online public opinion, a negative online public opinion can have a significant impact on social and public security if the evolution of its dissemination is not guided correctly and in a timely manner. Therefore, it is important to establish a prediction model for online public opinion, grasp the degree of risk and the trends of its development, and predict online public opinion crises in advance, in order to deal with them in a timely and effective manner to maintain the social order, improve people’s satisfaction and sense of security, and enhance the government’s governance system and capabilities.

According to Ren B. (2023) [4], methods for predicting public opinion on the Internet are divided into traditional mathematical prediction methods and nonlinear time-series prediction methods based on machine learning and deep learning. Based on Mu et al. (2024) [5], Time series data are obtained chronologically and illustrate the changing trend of the observed object over time, the prediction of the time series is a challenging and complex problem for researchers. Priyanto, S. et al. (2023) [6] used gray prediction and the exponential smoothing of traditional prediction models; Markov chains [7] have also been used. They are simple to apply but often have low prediction accuracy.

Prediction accuracy has significantly improved with the development of machine learning and deep learning. W. Wang et al. (2021) [8] designed a back-propagation (BP) neural network based on information granule for long-term prediction, which can facilitate the multiple steps forecasting in numerical level and improve the accuracy and efficiency for the long-term prediction. Huang Y., Chen F., and You D. (2018) [9] applied the optimization of a BP neural network using a mixture of a genetic algorithm and a particle swarm algorithm, thus improving the prediction effect of the model. Tiwari D. et al. (2022) [10] compares the result of Naive Bayes, Support Vector Machine (SVM) and Linear Regression in evaluating and predicting the future cases of Covid‐19, reveals that the prediction based on Naive Bayes is found to be more trustworthy.

In order to further improve the accuracy of online public opinion prediction and ensure the safety of social opinion, many scholars have adopted combined prediction and deep learning methods. Based on this research path, quite a number of relevant studies have acknowledged that LSTM outperforms other methods [11]. This also indicates that further steps to be taken are about selecting more capable hyperparameters for LSTM. Arbane, M. et al. (2023) [12] proposed an improved bidirectional-long short-term (Bi-LSTM) model and applied it to a database of Twitter posts about COVID-19 issues. Zhong Y. et al. (2023) [13] used a combination of logistic, ARIMA, and LSTM models to predict the evolutionary trends of Weibo (Microblog) opinions. LSTM can also adapt to the prediction of stock market public opinion by combining one-dimensional convolutional neural networks (1DCNN) [14]. Zhang W. et al. (2022) [15] tried to construct a CNN-LSTM model to describe the mechanism of the government information release (GIR) on the regulation of netizens’ negative emotions.

The Gray Wolf Optimizer is also frequently applied. Su Y. et al. (2023) [16] similarly focused on the evolution of public opinion, but they obtained their results by improving the Gray Wolf Optimizer, combining it with logistic and Lotka–Volterra models, a nonlinear function, an elite retention policy, and a Pareto-optimal solution, which enhanced the accuracy and universality of the model. To summarize, many scholars have studied the trends in the development of online public opinion from different perspectives, gradually extending these prediction methods, some of these literatures are concluded in Table 1 below.

**Table 1:** Models and performance metrics applied in literatures.

Reference	Year	Model	Performance metrics	Target of the study
Wang, W., Liu, W., & Chen, H.	2019	BP (Back propagation)	RMSE (Root mean square error)	To develop new prediction approaches for time series data based on information granule.
Huang Y., Chen F., and You D.	2018	BP	MRE (magnetorheological elastomers)	To develop a model for more accurate and stable prediction on changing trend of public opinion.
D. Tiwari, B. S. Bhati, F. Al‐Turjman, B. Nagpal	2022	Naive Bayes	MAE (Mean absolute error), MSE (Mean square error)	To evaluate and predict the future cases of Covid‐19.
Zhang, F., & Xia, Y.	2022	LSTM	RMSE, MAE, MAPE (Mean Absolute Percentage Error)	To forecast carbon prices.
Arbane, M., Benlamri, R., Brik, Y., & Alahmar, A. D.	2023	Bi-LSTM	MSE	Sentiment classification on social media platforms.
Zhong Y., He W., Zhang P., Zhang J., Lan X.	2023	Logistic-ARIMA-LSTM (LAL)	MAE, RMSE	To predict the changing trend of online public opinion.
Yi, J., Chen, J., Zhou, M., Hou, C., Chen, A., & Zhou, G.	2023	RNN-LSTM, 1DCNN	F1	To propose a framework to evaluate the stock market volatility.
Zhang, W., Li, L., Zhu, Y., Yu, P., & Wen, J.	2022	CNN-LSTM	R^2^	To propose a CNN-LSTM model for Chinese language sentiment classification.
Su, Y., Li, Y., & Xuan, S.	2023	improved multiobjective gray wolf optimizer (IMOGWO)	MRE, MAPE, R^2^	To predict the evolution trends of various types of complex public opinion.

However, data related to online public opinion have a certain time-series correlation, and it is difficult to mine correlations in time series data in the time dimension using statistics or traditional machine learning methods while maintaining sufficient accuracy; so, further research on the modeling and prediction of online public opinion using deep learning methods is indispensable.

Due to the changing trend of college online public opinion will be summarized to the discussion of time series, in this study, we introduce LSTM, a deep learning network, as the basis for a public opinion prediction model. Compared to other machine learning methods, LSTM has better performance on handling long-term dependency and tackling the gradient explosion and disappearance problems in long sequence predictions [17], the efficiency and the accuracy of the prediction applying LSTM can also be verified by numerous studies [5, 18]. So, we choose LSTM for its fine performance in processing time series and previous studies. Considering the performance of LSTM is nearly determined by its parameters, which are usually set empirically, the Gray Wolf Optimizer(GWO) is applied to enhance its training efficiency and prediction accuracy.

In conclusion, We establish an online public opinion risk model that pays more attention to information on risks related to public opinion and, at the same time, integrates public opinion risk index as an influencing factor, thus better reflecting the complexity and the extent of harm of online public opinion crises. On this basis, the parameters of the LSTM model were optimized by adding the Gray Wolf Optimizer, which improved the training speed and prediction accuracy of the model, allowing it to accurately predict the trend in the development of the heat and risk of online public opinion in emergencies to provide a basis for timely preventive measures.

The remaining sections of the article are below: Part 2 Methodology makes a brief introduction about basic principles of sentiment analysis, LSTM and GWO; In Part 3 we show the whole process of making a prediction through the model we propose and validation; In Part 4 we make an intervention experiment to manifest how the model can be applied by authorities and colleges in the reality. In Part 5, we give out our conclusion, limitations and future prospect of the study.

## 2. Methodology

### 2.1 Principles of the sentiment analysis model

SnowNLP is a Python library specializing in Chinese natural language processing. It comes with a positive and negative sentiment training set for Chinese and is well suited for the processing of Chinese text data; its principle is based on Naive Bayes [19].

It is assumed that there are two types of sentiment analysis: positive *A*_1_ and negative *A*_2_. A piece of an obtained comment contains a composition of mutually independent words, such as *B*_1_, *B*_2_…*B*_*n*_. The Bayesian formulas are as follows:

$$P(A_{1}|B_{1},\ldots ,B_{n})=\frac{P(B_{1},\ldots ,B_{n}|A_{1})\cdot P(A_{1})}{P(B_{1},\ldots ,B_{n})}$$ (1)

From the full probability formula,

$$P(B_{1},\ldots ,B_{n})=P(B_{1},\ldots .B_{n}|A_{1})\cdot P(A_{1})+(B_{1},\ldots ,B_{n}|A_{2})\cdot P(A_{2})$$ (2)

Substituting this into the above equation gives

$$P(A_{1}|B_{1},\ldots ,B_{n})=\frac{P(B_{1},\ldots ,B_{n}|A_{1})\cdot P(A_{1})}{P(B_{1},\ldots ,B_{n}|A_{1})\cdot P(A_{1})+P(B_{1},\ldots ,B_{n}|A_{2})\cdot P(A_{2})}$$ (3)

This equation is the basic computational formula for sentiment analysis with SnowNLP. The formula shows that the probability that a comment containing the words B_1_, B_2_…B_n_.has a positive sentiment can be calculated from a calibrated dataset.

### 2.2 LSTM

**Fig 1:**
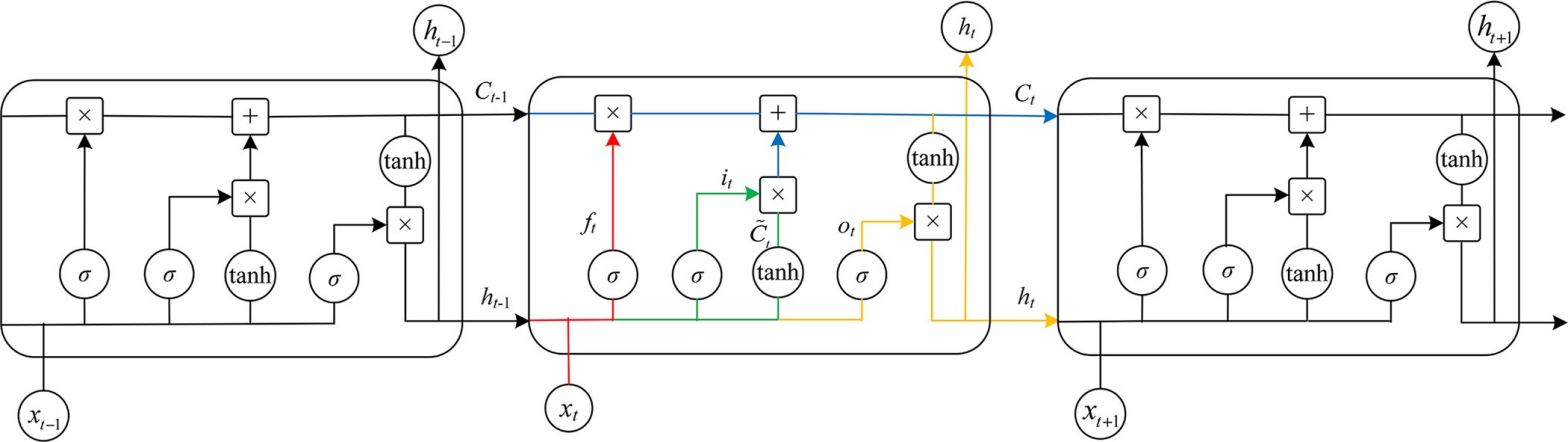


**Fig 2:**
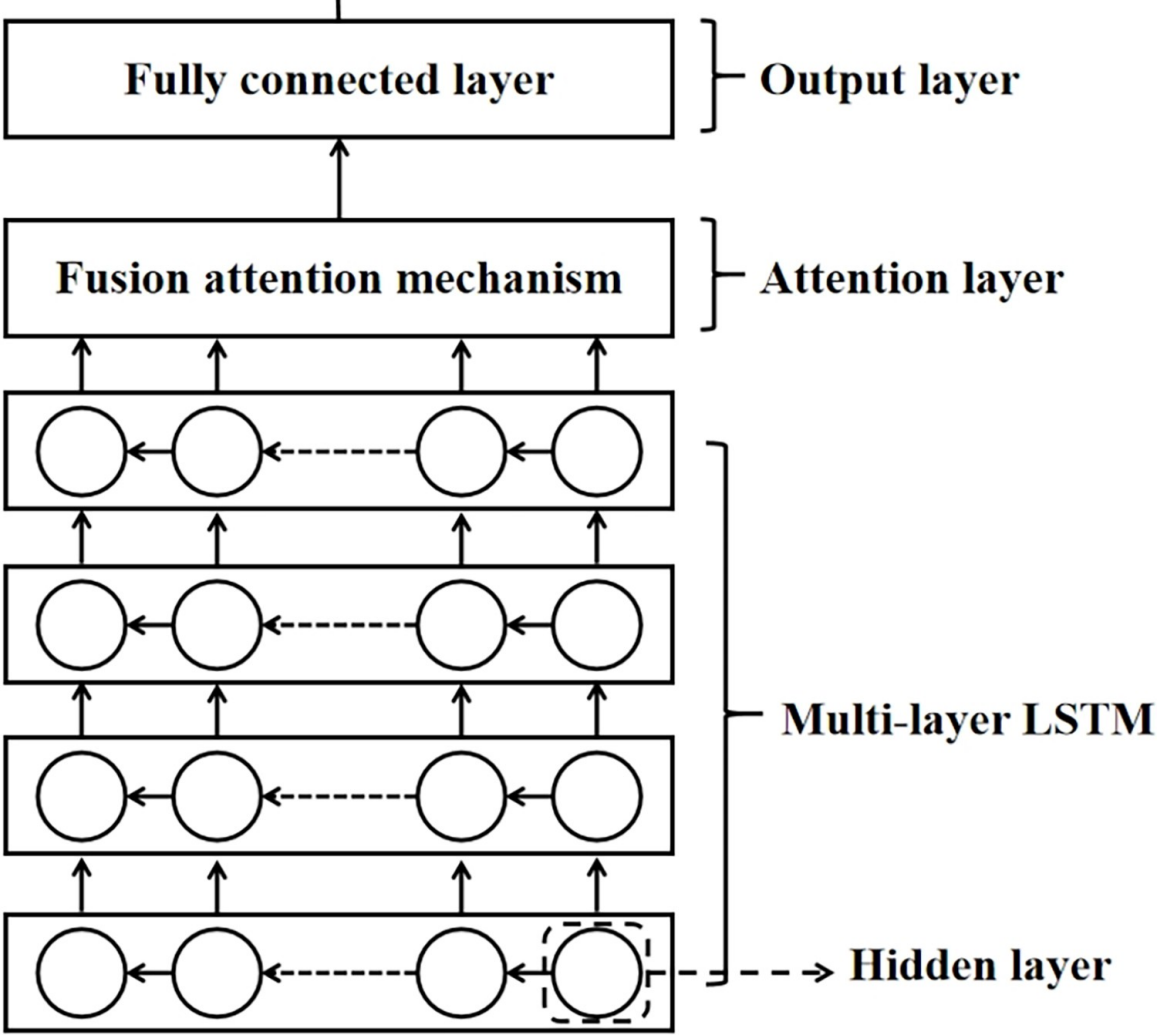


LSTM is adopted in deep learning and AI [20]. The LSTM approach has evolved to solve the vanishing gradient issue that might arise during training conventional RNNs [21]. LSTM works in basically the same way as a recurrent neural network (RNN) with a memory function; this advantage makes it possible to take the forward and backward dependencies of the input data into account and to output the results produced by the combination of network inputs and network history information at that moment, thus making it more suitable for modeling the trends of time series [22]. LSTM is not structurally different from an RNN, as both consist of an input layer, an hidden layer, and an output layer. The difference is that it solves the problems of vanishing gradients and exploding gradients [23] in the traditional RNN training process. The structure of LSTM we give in this study are shown in Figs 1 and 2 below.

**Fig 3:**
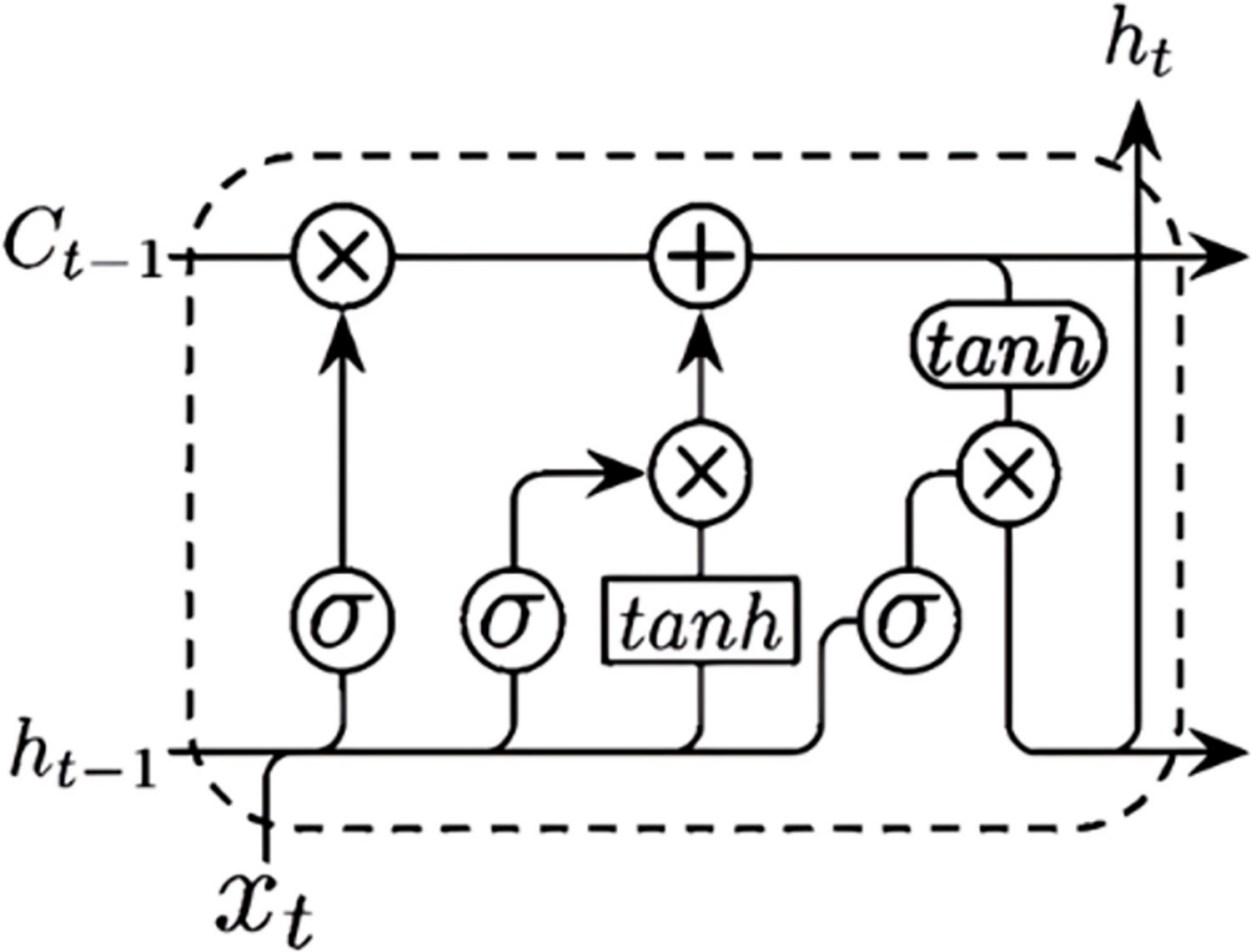


Unlike RNNs, the hidden layer of LSTM has memory units and gate structures, including input gates, output gates, and forgetting gates. Its constituent modules are shown in Fig 3. The hidden layer forward pass and gate structure control mechanism is as follows:

$$i_{t}=\sigma_{i}(W_{xi}x_{t}+W_{hi}h_{t-1}+W_{ci}c_{t-1}+b_{i})$$ (4)

$$f_{t}=\sigma_{f}(w_{xf}x_{t}+W_{hf}h_{t-1}+W_{cf}c_{t-1}+b_{f})$$ (5)

$$c_{t}=f_{t}ⓧc_{t-1}+i_{t}ⓧ\tan h(W_{xc}x_{t}+W_{hc}h_{t-1}+b_{c})$$ (6)

$$o_{t}=\sigma_{o}(W_{xo}x_{t}+W_{ho}h_{t-1}+b_{o})$$ (7)

$$h_{t}=o_{t}ⓧ\tan h(c_{t})$$ (8)

*x*_*t*_ is the input vector of the model, *h*_*t*_ is the output vector of the model, where it can be seen that the output vector of the previous layer participates in the construction of the network as the input of the layer, and *c*_*t*_ is the memory unit.

The hidden layer’s forward propagation and gate structure control mechanisms are as follows.

At moment *t*, the input gate updates the temporary value of the memory cell based on the hidden node *h*_*t*−1_ and the input data *x*_*t*_. The degree of state retention *C*_*t*_ at the previous moment determined by the forgetting gate is combined with the calculation of the input gate. The final state of the hidden layer is derived from the output gate, which has two parts; the first part utilizes the activation function *σ*_*o*_ to obtain the output state *o*_*t*_, while the second part consists of the composition of *C*_*t*_ after being processed by the activation function tan *h*. Finally, the output state *o*_*t*_ depends on the hidden layer state *h*_*t*−1_ at the previous moment and the input data *x*_*t*_ at the current moment.

Long-term dependencies in time series can be well captured and processed using LSTM models, as they can automatically and effectively learn important features and process internal correlations in data in response to the complexity of time series in the development of online public opinion.

### 2.3 The Gray Wolf Optimizer

The Gray Wolf Optimizer is an optimized search method inspired by the prey-hunting activities of gray wolves, which was proposed by S. Mirjalili et al. [24] in 2014 as a new meta-heuristic algorithm with advantages of strong global search ability and fast operation speed [25]. In nature, gray wolves survive by searching for food through collaboration and competition. The Gray Wolf algorithm takes advantage of this behavioral characteristic to find the optimal solution to a problem by simulating the positional transformations of the winners and losers of the gray wolves in the search space and gradually increasing the fitness value of the whole group.

The basic principle is the transformation of the position and iterative replacement based on the winners and losers among the members of the gray wolf pack. The algorithm consists of four basic steps:

1. Initializing the gray wolf population: the initial locations of gray wolves are determined, and their fitness values are calculated.
2. Search process: the position and fitness value of each gray wolf are updated according to the distance between the individual gray wolves and the fitness value. In this study, the model fitness function is the mean square error of the training set of the LSTM model.
3. Selecting the optimal gray wolf: the global winner, i.e., the optimal solution, is determined based on the fitness value.
4. Updating positions: the position of each gray wolf is updated based on the position of the optimal gray wolf and the relative positions of other gray wolves.

Since the Gray Wolf Optimizer was first proposed, different scholars have suggested various improvement strategies to balance the global and local searches and optimize the quality of solutions [16]. Initializing the individual positions of gray wolves with this method can increase the diversity of the group, which can improve the global search ability of the gray wolves to a certain extent and accelerate the convergence speed of the model. Secondly, parameter A in the GWO plays a key role in balancing its global search ability and local exploitation ability. The value of A in the classical Gray Wolf Algorithm varies linearly from 2 to 0 with the number of iterations. Mittal N. et al. (2016) [26] found that a nonlinear transformation of the parameter *A* contributes to a better search performance. In this study, the trigonometric cosine function was applied to nonlinearly vary parameter *A* in the algorithm.

The Gray Wolf Optimizer has the advantages of group collaboration, easy implementation, fast convergence, and powerful global search capabilities; it can converge to the optimal solution faster in high-dimensional optimization problems and search between multiple locally optimal solutions. In this study, the GWO was used to optimize the hyperparameters of the LSTM model and find its optimal solution.

### 2.4 Statement

We address that all terms and conditions for the source of the data are strictly complied during the process of data collection and analysis, we conducted whole research after consideration.

## 3. Prediction based on GWO-LSTM model and evaluation

This model was used to analyze and explore risky changes in the online public opinion of colleges and universities caused by online hot spots, based on the use of sentiment analysis and GWO-LSTM. The process included data collection, modeling, and risk index prediction. Rather than simply predicting discussion peaks related to public opinion, a public opinion risk index is introduced to describe the status of the public opinion. In order to demonstrate both the influence and the emotional inclination of online public opinion, we need to acquire the statistics of the comments and posts on the incident and the overall sentiment analysis of the discussion. In the study, web crawlers, Zhiwei data (these two for the statistics), and sentiment analysis are utilized to obtain the data. Then we use principal component analysis (PCA) [27] to extract the weight of two factors from the dataset and construct the public opinion risk index as the unified standard for gauging the risk of online public opinion risk. Applying the model to predict the variation trend of the public opinion risk index can better describe the status of the public opinion risk and make a more integral prediction of the trend.

**Fig 4:**
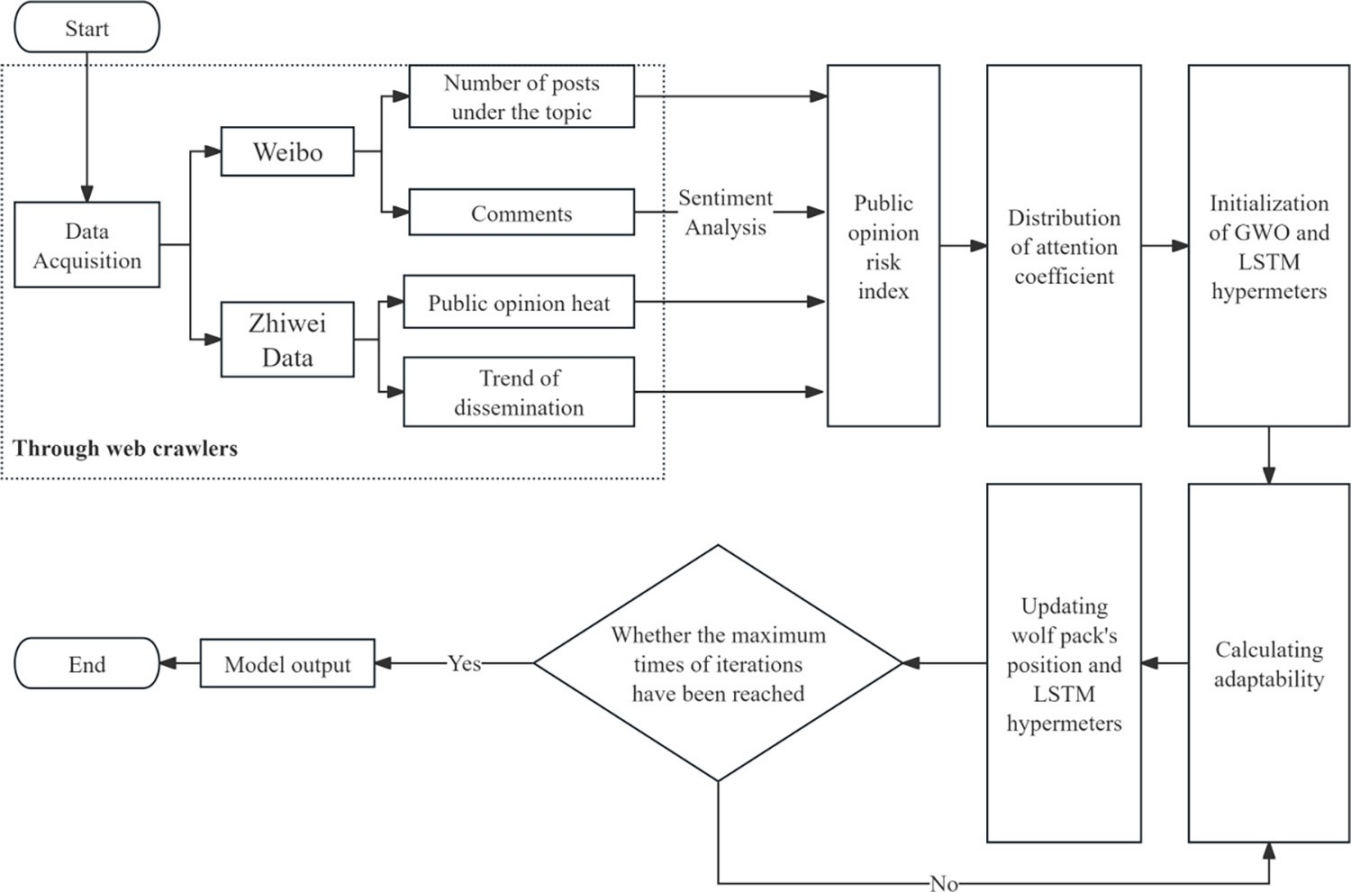


More specifically, The whole process of the analysis can be divided into three parts: data acquisition and preprocessing, prediction result, evaluation, and model comparison. Firstly, choose a specific online college public opinion incident as the research object and collect, and clean the relevant data. Secondly, utilizing the model to obtain the prediction results in the form of a public opinion risk index. Finally, using various evaluation criteria, and a comparison between the prediction and the real case to check the reliability. The specific process is shown in Fig 4.

### 3.1 Data acquisition and preprocessing

#### 3.1.1 Data acquired from Weibo and Zhiwei Data

The "Sichuan University Subway Incident" in June 2023 was taken as an example for constructing the model. Zhang Wei, a postgraduate student at Sichuan University, wrongly believed that a man on the Guangzhou subway had taken a picture of her without permission. They reached an agreement after the man showed her his phone to prove no photo was taken, but Zhang later still posted an image of the man on Weibo and accused him of invading her privacy. This irritated the man’s son and many other people; the image went viral, soon becoming the center of online discussion. The whole online event lasted for 16 days and 9 hours, during which many details, such as the initiation of the incident, the resolution of the incident (the man’s son accused Zhang of not obeying the conciliation contract and posting the man’s image online), and the announcement of Sichuan university was issued. The university’s response topped the trending topics on Weibo, where more than 620 million comments were launched. Zhang was accused of the invasion of privacy and having no respect for others, and the Sichuan University was also drawn into the mire for lack of moral education, both were condemned by netizens. This incident had a large-scale influence on social media and is strongly related to universities, which makes it a good example for the research on the evolution of college online public opinion risk.

The text sentiment analysis is crucial for attaining the public opinion risk index we proposed in this study, and the method we choose for the analysis is deep learning since this method can avoid the phenomenon of overfitting while processing a large amount of input data [17]. To understand and describe the public opinion of the "Sichuan University Subway Incident" better, we noticed that Weibo, which is similar to X (Twitter) and widely used among social media platforms in China, is an excellent platform for data acquisition, especially for its Weibo topic (similar to hashtags on Facebook) can collect numerous texts directly about the incident. By utilizing web crawlers, we chose a three-and-half-a-day period from the occurrence of this event as the dataset for model construction. The first dataset included comments under the Weibo topic "#The man proved that he did not sneak a photo but was accused online by the girl, who has apologized". After data cleaning, the number of valid comments was 6806, as Table 1 shows. In order to strengthen the explanatory power of the study, we decide to include the variation trend of the incident’s comments dissemination offered by Zhiwei Data, an independent third-party data assessment organization in China. It is included in the second dataset and shows the number of relevant comments and posts in a greater range. Both datasets can be described as public opinion heat, as higher “temperature” means more engaging the online discussion was at that time. Comments are all originally written in Chinese, we translated part of them from 1^st^ dataset into English and filled them into Table 2, and the full version can be found in S1–S3 Data in the Supporting information section.

**Table 2:** Weibo comments under selected 2 hashtags, from 1st dataset.

Serial Number	Commentaries	Timing(month-date-min-sec)
1	I’m sorry, I was wrong this time, but next time I’ll dare to do the same.	6–12 00:16
2	It’s too low a cost to cause a national negative impact on others, and if it’s all over with a simple apology, then this case will only encourage more people to continue to do so.	6–12 00:45
. . .	. . .	. . .
6806	The man can be forgiven because of kindness. Society cannot forgive because of justice. Otherwise, there will be more of such uncles being bullied for no reason. I would like to see this woman pay her due.	6–14 23:43

**Fig 5:**
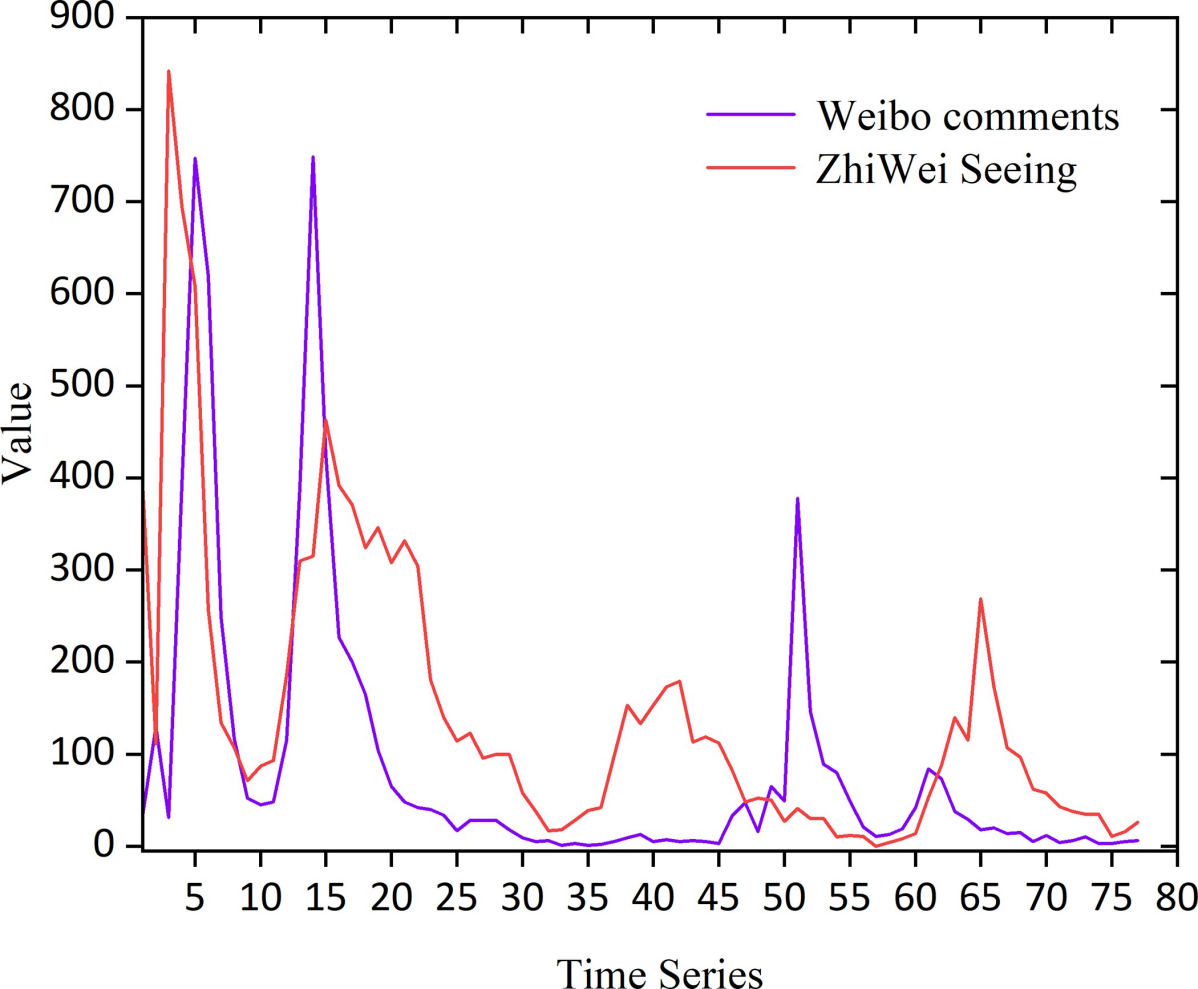


The moments of publication of the 6806 comments were counted for each hour, and the changes in the public opinion heat are shown in Fig 5, show the frequency of comments about the topic.

The collected time series started at 19:00 on June 11th, 2023 and ended at 24:00 on June 14^th^, 2023, lasting 77 hours in total. As shown from the changes every hour in the heat map of public opinion, there were four peaks of discussion during the three and a half days of this period, with the heat of public opinion reaching its peak 4 hours after the onset of the incident. Similar heat data trends were crawled and counted by both the Weibo and Zhiwei Data platforms. We adopted a combination of data sources and weighting to calculate the risk index for online public opinion to make the model more generalized and accurate.

Normalization. Considering the evaluation systems and units of data obtained from different platforms, the time series data were linearly normalized to eliminate the influence of the range of the scale and data values. The same formula was also used for the statistics from the sentiment analysis in this study. The formula is as follows:

$$x^{\prime }=\frac{x-\min\left(x\right)}{\max\left(x\right)-\min\left(x\right)}.$$ (9)

#### 3.1.2 Data from sentiment analysis

Sentiment analysis was used to mark, categorize, and count the number of negative comments among the 6806 valid comments using SnowNLP [19], as Table 3 shows.

**Table 3:** Emotion analysis of Weibo comments.

Emotion	Negative	Positive or Neutral
Number	5210	1596

Statistics of the time at which comments were posted were used to calculate the average sentiment score per hour and to obtain the change in the sentiment of the comments in the time series of public opinion, which was used as an important indicator of the public opinion risk index.

**Fig 6:**
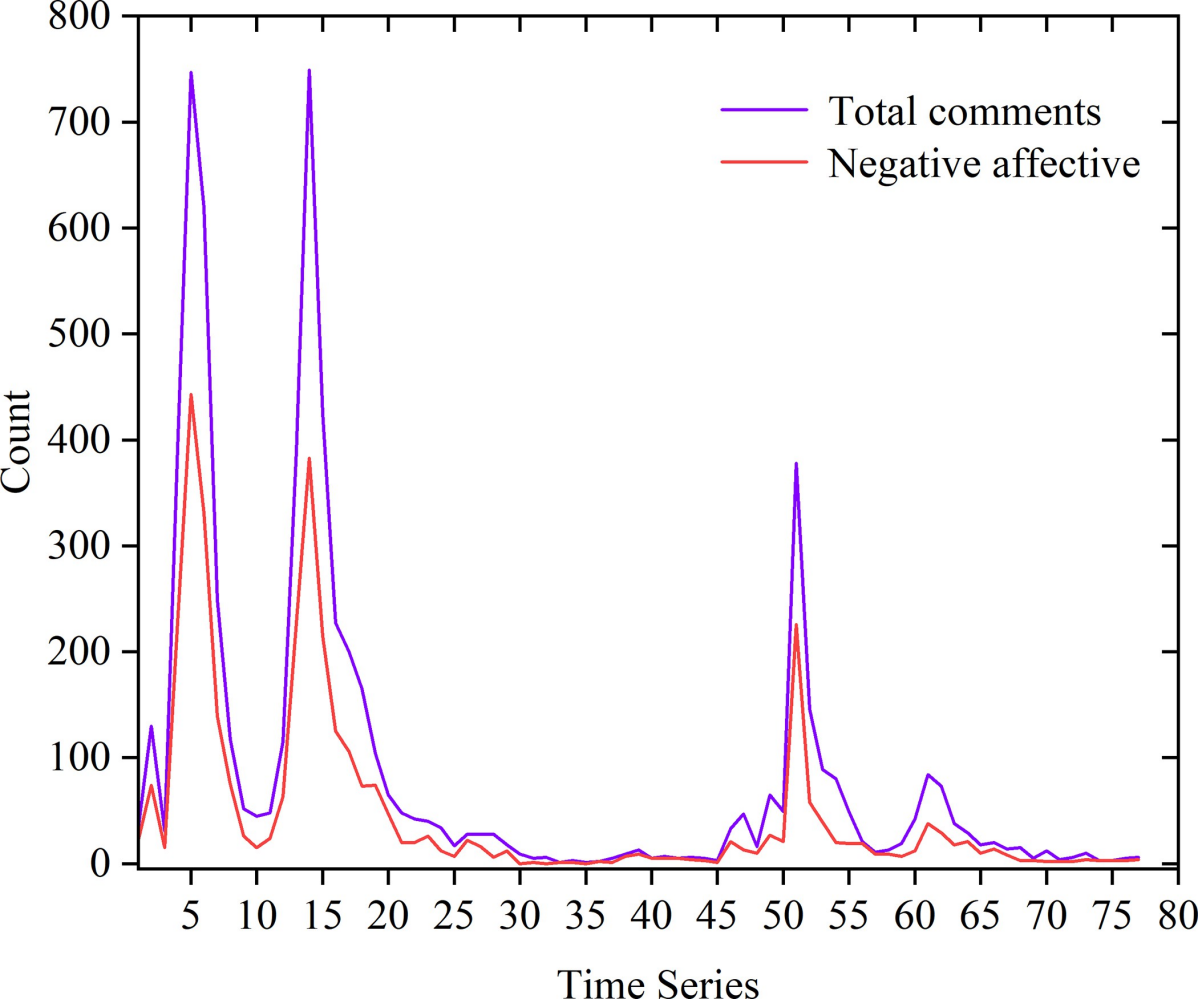


The difference between the number of negative comments and the number of remaining (positive and neutral) comments in an hour was used as the degree of negative sentiment for a comparison, as Fig 6 shows. From the analysis, it can be seen that the number of negative comments was always larger than the number of positive and neutral comments during the selected period in which public opinion on this topic developed; this indicates that the online public opinion of these events was characterized by negative emotions. The trend of the degree of negative emotion was generally consistent with the total comments; when the total number of comments was higher, the degree of negative emotion decreased, and when the total number of comments reduced, the degree of negative emotion decreased, indicating a positive correlation between the degree of emotion and the heat of online public opinion.

### 3.2 Prediction result

#### 3.2.1 Public opinion risk index

There is no standardized and generally accepted definition of a public opinion risk index; this study considered both data sources mentioned above while adding statistics from sentiment analysis. We consider defining an opinion risk index using a methodology that weights the data in such a way that on the one hand the different weighted values reflect the importance of that data source (or indicator) and on the other hand it is mathematically simple and reliable. The defined index is intended to reflect the riskiness of public opinion at the current moment or the threat to the coming. And from the construction of this index, it can be used as an important reference for controlling the development of public opinion.

We used principal component analysis (PCA) to perform an interpretation of the importance of the three statistical indicators [28] to obtain the weights for the evaluation model—shown in Table 4—and then used the following formula with the weights to calculate the public opinion risk index.


Z=W⋅A (10)

**Table 4:** Weights of data resources.

Data sources	Zhiwei Data trends	Sina Weibo comments Short-lived enthusiasm	Sina Weibo comments Negative emotion
**Weights *ω***_***i***_	0.383	0.263	0.354

**Fig 7:**
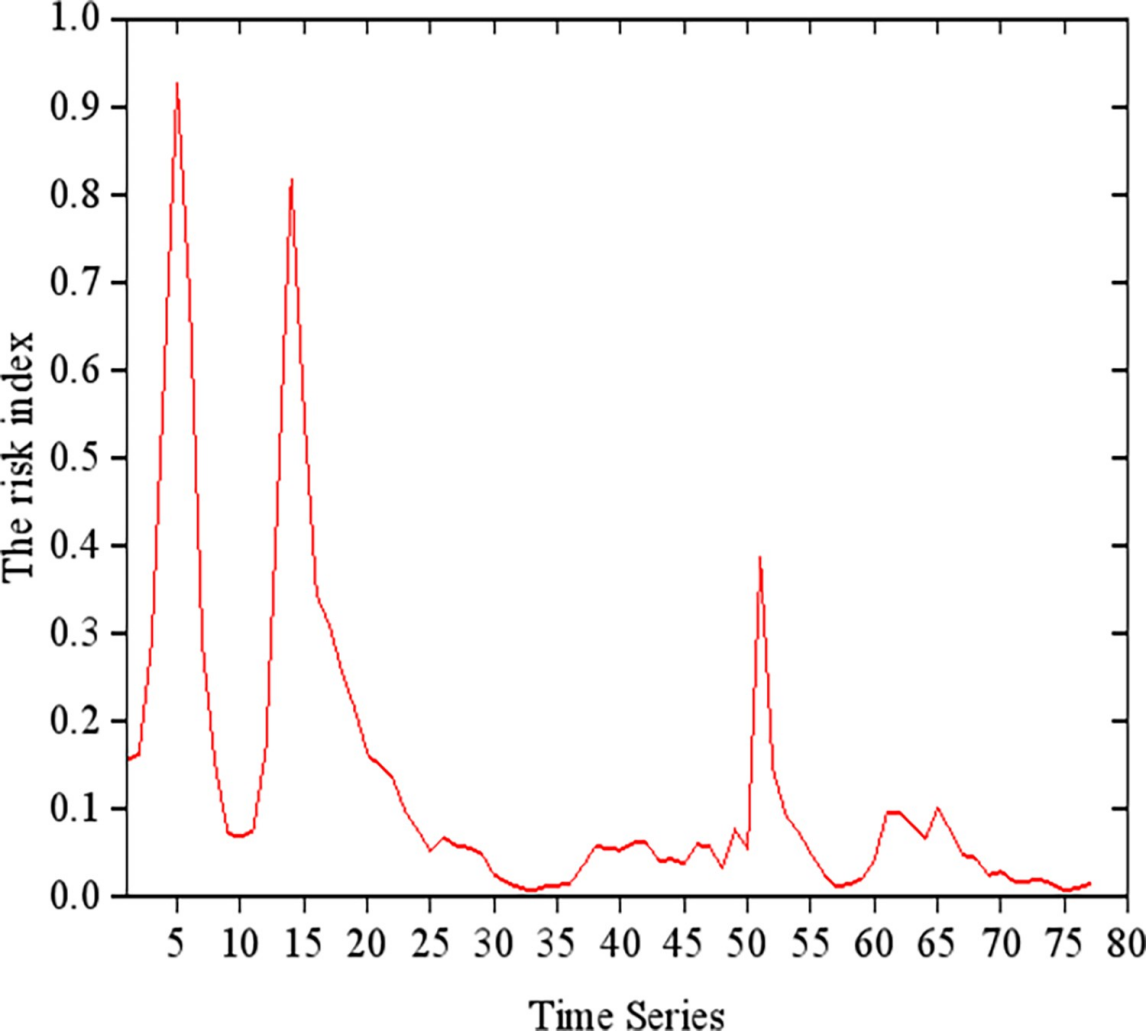


The advantage of this method is that information on the heat of public opinion was included and both positive and negative aspects of public opinion were obtained by combining that information with the results of the sentiment analysis. Using this formula and the data we acquired in the former process, we can calculate the changing trend of the index during the time period we selected before and it is shown in Fig 7 below.

The time series show how long had been since the outbreak of the event, and the closer the index is to 1, more intensively it was discussed by netizens and then the public opinion risk is higher, more severely the university was accused. In Fig 7, public opinion reached the maximum risk value five hours after the outbreak and the obvious periodicity exists since less people commenting in the night.

#### 3.2.2 Prediction based on GWO-LSTM

**Fig 8:**
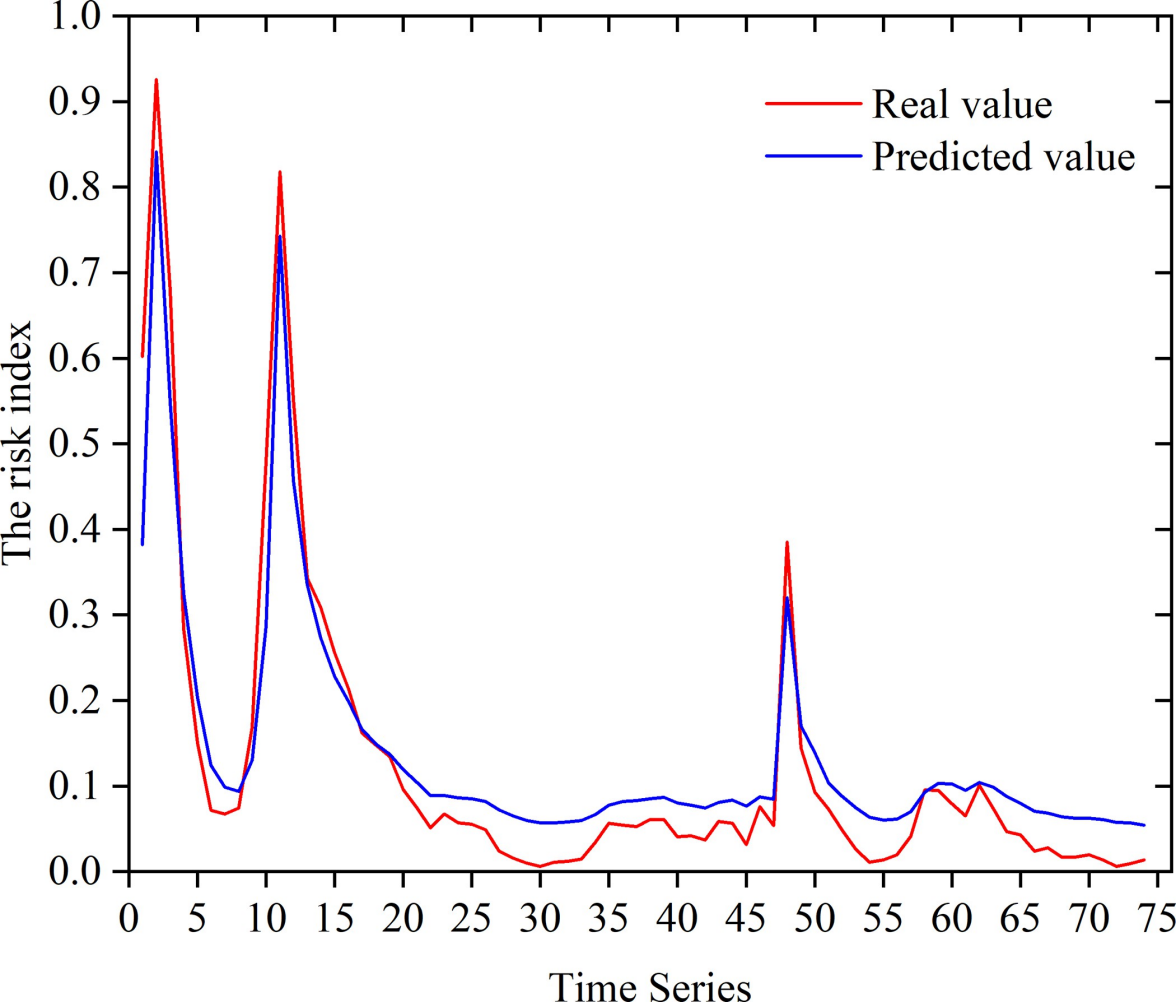


We used the TensorFlow framework in a Python environment to build an LSTM neural network, adding the fusion attention mechanism to obtain the best model results [29, 30]. According to the risk index model for online public opinion constructed in this research, 77 risk indices in time series were put into the model for training. Then, 75% of the indices were allocated for the training set, and the rest were allocated for the testing set. Finally, the overall development of public opinion was predicted; a chart depicting a comparison between the predicted values and the actual values is shown in Fig 8, and the prediction error of the testing set is shown in Table 5.

**Table 5:** Error comparison.

Time series	Actual value	Post-optimization	Relative error
64	0.04662	0.04487	3.75%
65	0.04282	0.04051	5.39%
66	0.02379	0.02489	4.62%
67	0.02791	0.02861	2.51%
68	0.01675	0.01884	12.48%
69	0.0168	0.01752	4.29%
70	0.0197	0.02032	3.15%
71	0.01374	0.0145	5.53%
72	0.00624	0.00568	8.97%

### 3.3 Evaluation and model comparison

#### 3.3.1 Evaluation criteria

To evaluate the prediction accuracy of the prediction model, representative statistical indicators, such as the R^2^, MSE, MAE, RMSE, and the average relative errors, were selected. The formulas of these criteria are shown below:

$$R^{2}=1-\frac{\sum_{i}{\left(\hat{y_{i}}-y_{i}\right)}^{2}}{\sum_{i}{\left(\bar{y_{i}}-y_{i}\right)}^{2}}$$ (11)

$$MSE=\frac{1}{m}\sum_{i=1}^{m}{\left(y_{i}-\hat{y_{i}}\right)}^{2}$$ (12)

$$MAE=\frac{1}{n}\sum_{i=1}^{n}\left|y_{i}-\hat{y_{i}}\right|$$ (13)

$$RMSE=\sqrt{\frac{1}{n}\sum_{i=1}^{n}{\left(y_{i}-\hat{y_{i}}\right)}^{2}}$$ (14)

The criteria that are close to 0 indicate better performance, and the evaluation criteria results of GWO-LSTM and rest 4 models are shown below.

In the Table 6 above, it can be seen that the introduction of the GWO–LSTM model provided better performance. Among many prediction models, the comparison of 4 evaluation criteria clearly indicates GWO-LSTM as the optimal model. In addition, compared to classical LSTM, GWO combined LSTM shows obvious decrease among 4 criteria, this result manifests the necessity of applying GWO.

**Table 6:** Comparison of evaluation criteria.

MODEL	R^2^	MSE	MAE	RMSE
PSO-BP	0.0107	0.0032	0.0107	0.0111
SVR	0.0088	0.0016	0.0088	0.0103
CNN	0.0080	0.0025	0.0080	0.0085
LSTM	0.0079	0.0012	0.0079	0.0086
GWO-LSTM	0.0041	0.0006	0.0041	0.0044

#### 3.3.2 Model comparison

**Fig 9:**
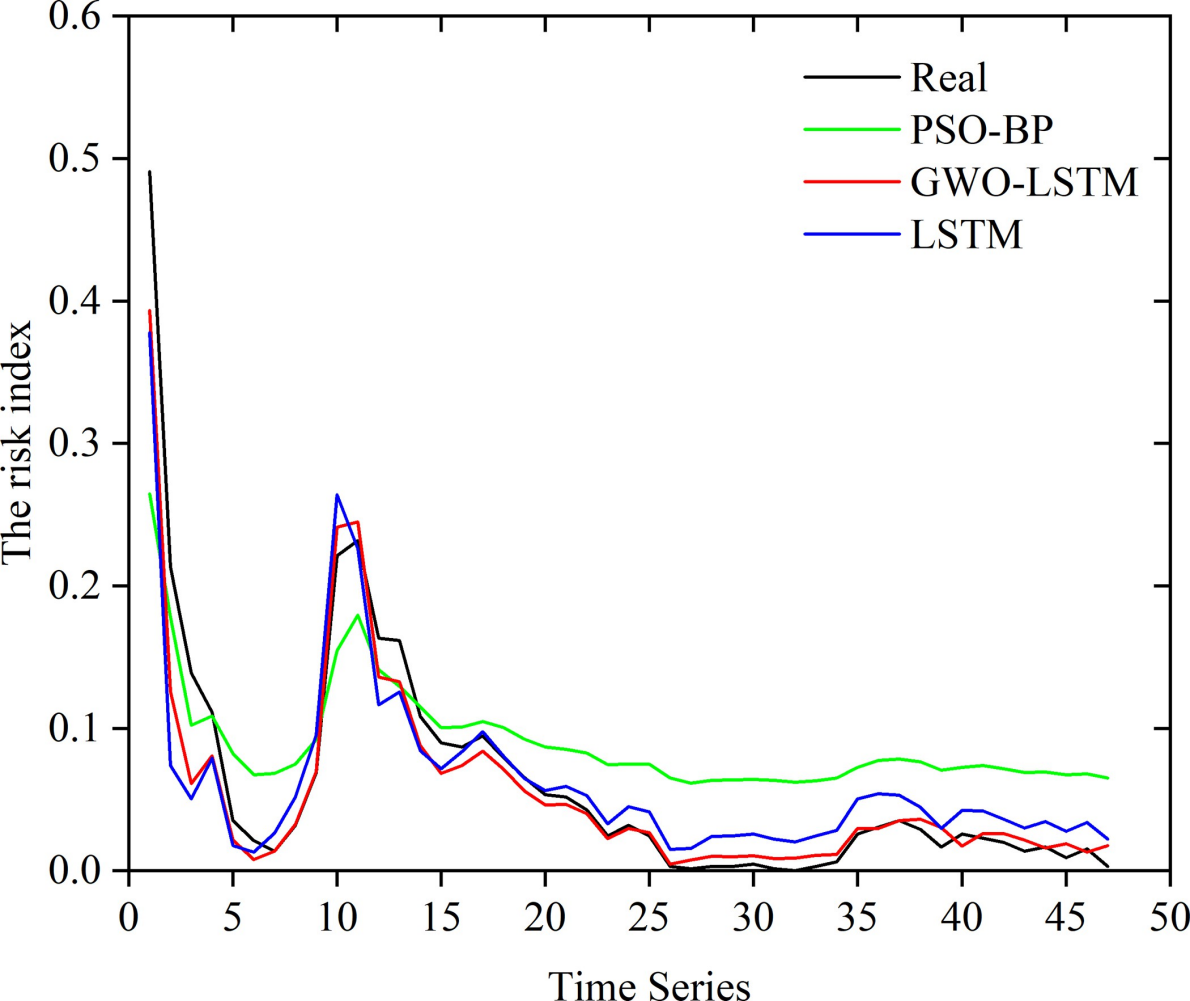


We select another Weibo topic related to this incident: "#the Sichuan University student involved in the subway incident was put on probation" to verify the accuracy of the proposed model. The results are shown in Fig 9. For detailed prediction data refer to S4 Data.

In the graph, black indicates the actual value, and red indicates that of the optimized LSTM model, which was the closest, with an average absolute error of 13.8%. It can be seen that both the BP neural network and the LSTM neural network could relatively accurately predict the trend of public opinion in the test time series, but there was a large gap between the value predicted by the PSO–BP network model and the actual value, while the prediction model using GWO-optimized LSTM had the best prediction accuracy, as its prediction was almost the same as the actual value. This indicated that the LSTM neural network had good adaptability for time-series prediction, and, when combined with the GWO, the LSTM model had better training and higher prediction accuracy than those of the LSTM model with conventional parameters. Compared with traditional methods, the model-training method applied here had high prediction accuracy, good prediction stability, and smaller fluctuations, and it could better fit the changes in actual online public opinion.

## 4. Intervention experiment

It is not practical to only offer a forecast of the variation trend of the risk index, as a useful tool we aim to propose here for authorities and colleges to take measures to tackle public opinion crises, applying this model to an intervention experiment and giving instructions based on the prediction for authorities and colleges is necessary.

**Fig 10:**
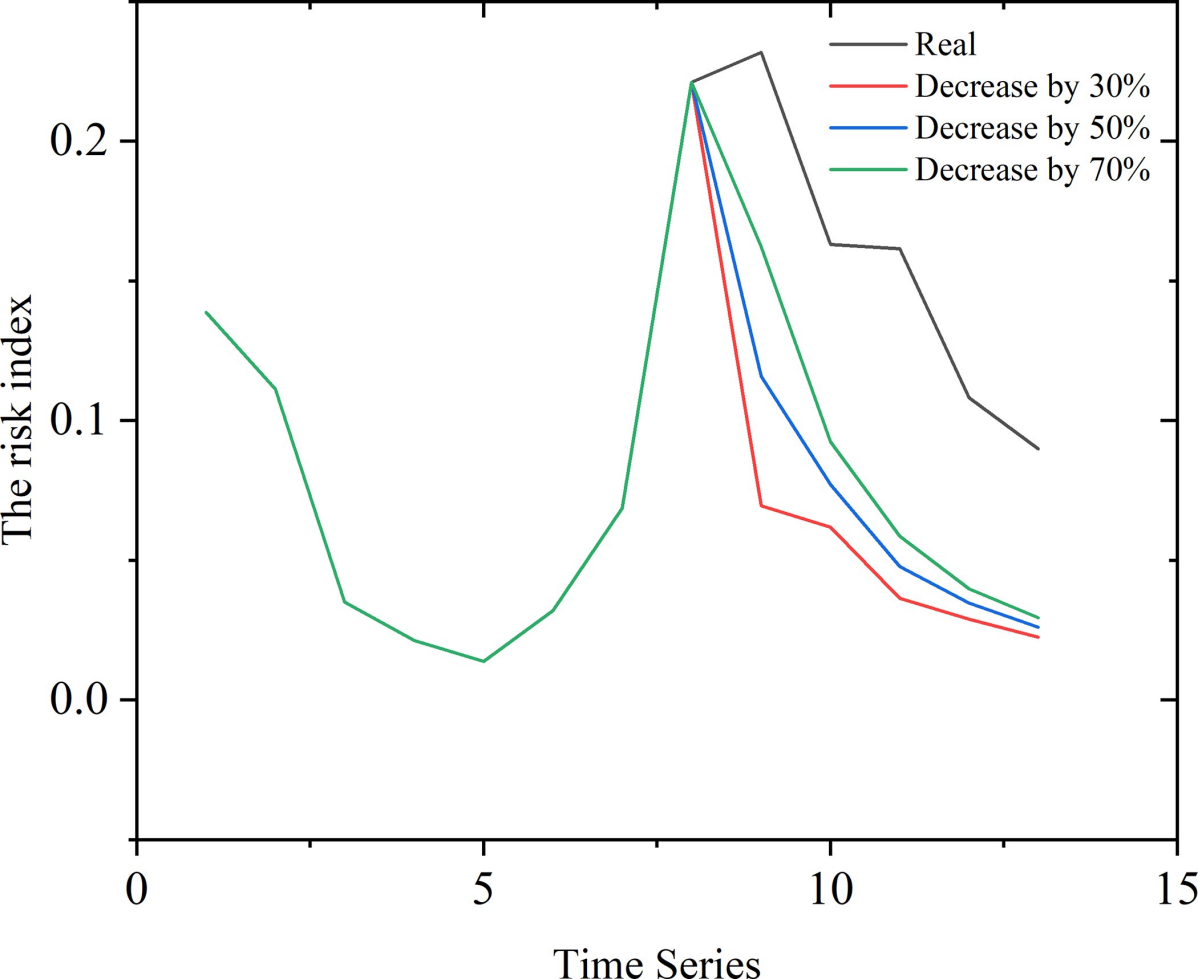


In order to use the model for the analysis of the evolution of events related to public opinion on campus, artificial interventions at specific points of time during the events were launched. We intervened at the second peak of the sequence, which meant that the risk was intentionally lowered by 70%, 50%, and 30%. The results are shown in Fig 10.

We launched the intervention at the second peak to simulate the real case that universities failed to realize the threat from the event and did not address it until the second peak. The content of the intervention can be the announcement from authorities, response, and control to radical comments, etc. From the chart of the analysis of the evolution with the validation set shown in Fig 8, it can be seen that, in the case of human intervention to reduce the risk value of the heat of public opinion, the subsequent development of public opinion tended to stabilize more quickly. Moreover, the results showed that there was not much difference in the smooth development of the subsequent developments when the risk index was reduced by 30% and 70%. However, if the risk index was only reduced by 30%, the subsequent development of the situation had a larger impact than if no intervention was made. This showed that the prompt intervention in, and guidance of, public opinion had a certain effect on the overall development of public opinion, and if public opinion is not controlled in time or is managed using a laissez-faire policy, this could result in great losses of social benefits and other problems.

## 5. Conclusion, limitations and future prospect

### 5.1 Conclusion

This study focused on the concepts of risk and sentiment analysis as important considerations when predicting changes in the public opinion of universities and used LSTM as a method for predicting the trend of a public opinion risk index to analyze the evolution of public opinion risk in the case of human intervention. By applying evaluation indexes like R^2^, MSE, MAE, and RMSE, the GWO-LSTM model proposed here is verified to outperform other models and offer more accurate prediction results. Validation and evolutionary analysis of the model were also conducted by using recent events related to online college public opinion, and the final prediction results were found to be consistent with the actual results, while the results on the evolution of public opinion revealed the importance of prompt intervention. In summary, this study focuses on college online public opinion as a specific field, proposes and verifies a variation prediction model on online public opinion, which is supposed to make a difference in public opinion regulation. The progress is valuable in both the academic field and practical management work, making the study significant.

### 5.2 Limitations and future prospect of the study

There are still several limitations in this study. Based on these limitations, the prospect can be given to improve the quality of further studies and expand the scope of the research field in the future. Firstly, the quality of the dataset obtained from Weibo might be affected by the existence of troll factories and the regulations on comments. As numerous factors affecting the evolution and spread of online public opinion, more multiple data resources can improve the quality of validation and simulation process. Secondly, more evaluation indexes can be added to check the reliability of the model, like VAF, IOA, IOS, and a-20 [18, 31–33]. Lastly, since the hyperparameters of LSTM are manually set, it is always crucial to focus on the latest studies for more capable hypermeters.

## Supporting information

S1 Data This is the data of comments and posts obtained through web crawlers (in Chinese) and the preprocessing. (XLSX)

S2 Data This is the data of comments and posts obtained through web crawlers (in Chinese) and the preprocessing. (XLSX)

S3 Data This is the data of comments and posts obtained through Zhiwei data. (XLSX)

S4 Data The data we used to calculate public opinion risk index. (XLSX)
